# Preventive and Protective Effects of Nicotinamide Adenine Dinucleotide Boosters in Aging and Retinal Diseases

**DOI:** 10.3390/ijms262210923

**Published:** 2025-11-11

**Authors:** Saba Noreen, Soon Sung Lim, Deokho Lee

**Affiliations:** 1 Department of Food Science and Nutrition, Hallym University, 1 Hallymdeahak-Gil, Chuncheon 24252, Republic of Korea; d21505@hallym.ac.kr; 2 Korean Institute of Nutrition, Hallym University, 1 Hallymdeahak-Gil, Chuncheon 24252, Republic of Korea

**Keywords:** retinopathy, B vitamins, nicotinamide mononucleotide, nicotinamide, sirtuins, oxidative stress, inflammation, mitochondrial function, neuroprotection

## Abstract

Nicotinamide adenine dinucleotide (NAD^+^) boosting can sustain energy metabolism and neurovascular stability in the retinal tissue. Depletion of NAD^+^ is linked to the development of pathological retinal conditions, such as age-related macular degeneration (AMD) and diabetic retinopathy (DR). Mitochondrial dysfunction, oxidative stress, and inflammation occur in these diseases. This review summarizes substantial evidence of therapeutic NAD^+^ boosters, including nicotinamide, nicotinamide mononucleotide, or nicotinamide riboside. They help improve mitochondrial function and lessen neurovascular injury. We also emphasize the importance of natural products and sirtuins in facilitating cytoprotective effects through the regulation of mitochondrial balance and inflammation. Developments in drug delivery methods, such as nanoparticle encapsulation and targeted eye treatments, are promising for enhancing the bioavailability and effectiveness of NAD^+^ boosters. The novelty of this work is its combination of mechanistic insights regarding NAD^+^ metabolism with efficacy data from preclinical studies. Furthermore, natural products may work together to boost their therapeutic effects against retinal damage. Together, our review article highlights NAD^+^ metabolism as a potential therapeutic target for addressing retinal degeneration and maintaining vision in aging, neurologic disorders, and various metabolic diseases, including diabetes.

## 1. Introduction

The retina is notable for being one of the most metabolically active tissues in our body. This elevated metabolic rate stems from the necessity for proper phototransduction while also sustaining ionic gradients and neurotransmitter flow essential for processing visual data [1,2,3]. Photoreceptors (rods and cones) synapse with bipolar cells. They are modulated by horizontal cells. Bipolar cells contact retinal ganglion cells (RGCs) and amacrine cells. Then, RGCs project their axons to the brain for the visual data. The energy demands of the retina can be satisfied by mitochondrial oxidative phosphorylation, a critical mechanism for ATP production, although it is not the whole mechanism for production. Dysfunctions in this metabolic system can trigger a detrimental sequence marked with oxidative stress, inflammation, and, eventually, retinal neuronal loss in age-related macular degeneration (AMD), diabetic retinopathy (DR), glaucoma (damage to RGCs), or different ischemic retinopathies, depending on the cell types in the retina [4,5,6].

Nicotinamide adenine dinucleotide (NAD^+^) is essential for retinal energy metabolism and the cellular reaction to various stress types. This can be related to direct redox reactive oxygen species (ROS), hypoxia, or ischemia/reperfusion stress. In addition to its established function as a redox cofactor in glycolysis, the tricarboxylic acid cycle, and the mitochondrial electron transport chain, NAD^+^ is important for the functioning of essential signaling enzymes, such as sirtuins (SIRTs), poly (ADP-ribose) polymerases (PARPs), and NAD^+^, utilizing glycol hydrolases, such as CD38 [7,8,9]. These enzymes influence numerous cellular functions, including DNA repair, chromatin alteration, immune response, and cell longevity [10,11,12]. Mitochondrial energy metabolism is highly dependent on NAD^+^ as a cofactor. Furthermore, mitochondrial and cytosolic NAD^+^ metabolism are linked via redox reactions.

The retina can regulate NAD^+^ concentrations through various pathways, with enzymes such as nicotinamide phosphoribosyl transferase (NAMPT) and nicotinamide mononucleotide adenylyl transferases (NMNATs) serving critical roles in its synthesis and circulation. Nicotinamide mononucleotide (NMN) and nicotinamide riboside (NR) enhance NAD^+^ biosynthesis, having health benefits. NMN is synthesized from NAM by the rate-limiting enzyme, NAMPT. NMN is synthesized from NR through an NR kinase-mediated phosphorylation reaction. NMN is converted into NAD^+^ by NMNATs.

Interruption of those pathways, whether by genetic deletion, pharmacological blockade, or injury, has been experimentally shown to lead to swift degeneration of retinal neuronal or non-neuronal cells, failure of mitochondrial function, and significant visual deficits and vision loss [13,14,15,16,17].

NAD^+^ levels are now known to decline with age in various tissues, such as the retina and central nervous system (the brain or the spinal cord). This decrease is caused not only by lower expression of enzymes associated with biosynthesis but also by dysfunctions of enzymes such as PARPs, CD38, and sterile alpha and toll/interleukin-1 receptor motif-containing 1 (SARM1), particularly under oxidative or inflammatory stress situations [18,19,20]. The ensuing NAD^+^ shortage worsens mitochondrial impairment, increases ROS generation, disturbs metabolic balance related to lipids and glucose, and induces a mild inflammatory condition known as “inflammaging”. This inflammaging plays a major role in the degeneration of retinal tissues and also causes damage to the optic nerve and cornea in aging eyes [21,22,23]. Regarding inflammation and aging, links between microglial activation and NAD^+^ depletion have also been discussed in several studies [24,25].

Due to the essential function of NAD^+^ in supporting retinal health, studies have increasingly concentrated on methods to enhance NAD^+^ availability. This encompasses oral or systemic addition of NAD^+^ precursors like nicotinamide (NAM: a vitamin B3 derivative), NMN, and NR, along with the inhibition of NAD^+^-depleting enzymes [18,20,26,27]. These therapies can restore retinal NAD^+^ levels, boost cell survival pathways, and modulate neurovascular degeneration.

## 2. AMD and NAD^+^ Boosters

AMD is considered one of the primary reasons for visual impairment and vision loss in older individuals. Developed countries have many AMD cases. Although visual impairment is not extensively detected at the initial stage of AMD, patients can experience a greater degree of visual impairment, including color perception dysfunction or contrast sensitivity reduction at the later stage of AMD, as the outer retina is gradually degenerated. Aged eyes can have an accumulation of cellular debris (called “drusen”) in the region of the interface between the RPE and/or the choroid [28]. Its debris contains various types of proteins, lipids, carbohydrates, and other components. When its volume increases over time, transport of important nutrients to the RPE or waste products to the choroid can be disrupted. During this process, oxidative stress, inflammation, and pathologic angiogenesis can occur. Pathologic angiogenesis (called “choroid neovascularization” or “macular neovascularization”) in the eye is a severe condition of malformed blood vessels, causing the RPE and outer retinal cells (especially photoreceptor cells) to be damaged, leading to visual impairment and vision loss, if untreated.

Animal models have been widely developed in research for AMD [29,30]. Frequently utilized species comprise rodents, which are genetically manipulable, inexpensive, and beneficial for mechanistic and genetic research. Rabbits are primarily used for pharmacokinetic and drug delivery studies. No single model captures the entire range of human AMD, but each provides unique research benefits: rodents for studying molecular mechanisms, primates for neovascular AMD research close to humans, pigs for their anatomical resemblance, and rabbits for studies involving delivery, necessitating careful interpretation of preclinical findings. There are continuous attempts to enhance predictive validity.

Animal models for AMD offer important insights into the mechanisms of human AMD conditions. However, they have limitations for translation. Basically, metabolic factors with aging are difficult to handle in animal models. Macula points are even limited for rodent models. Furthermore, the research budget for the use of non-primates is cost-inefficient. Therefore, more efforts are needed to address this issue.

NAD^+^ boosting and AMD treatment have been linked with several studies. Ren et al. found that NMN ameliorates RPE senescence and inflammation [31]. In their study, RPE cell senescence and damage were induced by sodium iodate (NaIO_3_) treatment. They found that ROS may be associated with the NaIO_3_-induced ARPE-19 cell senescence. Since the in vitro system is highly limited to mimic the entire physiological characteristics of the RPE cells, they further confirmed that NMN could exert anti-senescence effects in vivo, histologically, and biochemically.

Du et al. found that reductive carboxylation might be a main metabolic pathway in the RPE. Furthermore, supporting its carboxylation by supplementations with an NAD^+^ precursor could protect against RPE damage under oxidative stress conditions [32].

Saini et al. demonstrated that NAM suppresses AMD’s pathologic biomarkers, such as drusen components and an angiogenesis factor (especially, VEGF), further modulating levels of complements and inflammatory molecules, finally supporting RPE preservation [33]. The model used in this study was human-induced pluripotent stem cells (iPSCs) of AMD.

Jadeja et al. summarized that targeting NAMPT and NAD^+^ biosynthesis can modulate RPE senescence in aging and/or degenerative ocular diseases, where RPE dysfunction is highly involved [34].

Zhu et al. showed that NR mitigates retinal damage by suppressing damaged DNA-stimulated microglial activation and pyroptosis [24]. Balb/c mice were used to receive extensive light exposure to cause retinal neuronal damage and loss. This stress is related to mitochondrial dysfunction and the release of dsDNA. NR treatment suppressed this pathological process. Based on this study, outer retinal damage can be modulated by NR administration.

Taken together, modulation of NAD^+^ metabolism is crucial to prevent or protect against AMD-related pathologic processes. NAD^+^ precursors can restore the balance of cellular redox status and activate the SIRT pathways by improving mitochondrial function, reducing oxidative and inflammatory stress. This can maintain the integrity of retinal neurons and retinal vasculature.

## 3. DR and NAD^+^ Boosters

DR remains the most prevalent microvascular complication associated with diabetes and ranks as a major global cause of vision impairment, affecting approximately one-third of diabetic individuals worldwide [35,36,37]. The burden of DR not only includes visual impairment, but also physical, emotional, and socioeconomic challenges [38]. Several key features of DR present pericyte loss, microaneurysm formation, the increased blood–retinal barrier (BRB) permeability, and retinal neovascularization. Those pathophysiologic outcomes are highly interconnected. Proliferative DR (PDR) is defined by the occurrence of retinal neovascularization, which is an advanced form of DR. Retinal neovascularization is induced by increased VEGF levels in the eye. This mechanism has been known to be involved with activations of hypoxia-inducible factors (HIFs; especially, HIF-1alpha) [39,40,41].

The role of oxidative stress in DR also highlights its central contribution to retinal damage through excessive ROS generation. ROS generation can also be affected by HIF activation in retinal neuronal cells. Key pathological features such as mitochondrial dysfunction, retinal cell apoptosis, chronic inflammation, and lipid peroxidation, driven by metabolic pathways, including the polyol pathway, protein kinase C (PKC) activation, and advanced glycation end-products (AGEs), are seen in DR [42]. Antioxidant defenses may be needed using promising therapeutic strategies involving natural antioxidants like polyphenols and carotenoids, as well as the activation of protective pathways, including antioxidant NRF2 and SIRTs. In this respect, NAD^+^ metabolism might be involved in the neuroprotective effects against DR-mediated retinal damage, although understanding cell-type-specific mechanisms is highly required.

Jung et al. [43] explored the function of NAM in protecting against diabetic retinal neurodegeneration in rats. They administered streptozotocin (STZ) to male Sprague–Dawley rats to induce diabetic conditions, followed by oral NAM administration at 500 mg/kg/day for either 4 or 12 weeks. The NAM therapy lowered oxidative stress and oxidative DNA damage in ocular tissues (the inner retina, including RGCs) and diminished reactive gliosis, as shown by decreased glial fibrillary acidic protein expression. The research found that NAM supplementation may reduce inner retinal neurodegeneration in diabetic retinas by mainly lowering oxidative DNA damage and improving DNA repair processes.

Although the retina has not been directly examined, NMN’s therapeutic potential has been examined in type 2 diabetes by Popescu et al. [44]. They suggested NMN can improve glucose uptake from the bloodstream in high-fat diet mice. The thermogenesis pathway can be upregulated by NMN in muscle tissues. Furthermore, NMN can stimulate adipose cell proliferation. Yamane et al. found that oral NMN administration can increase plasma NMN and insulin levels in humans [45]. As ocular damage is highly related to the other organs in systemic metabolic diseases, NMN’s various roles in tissues can be used to affect retinal function under stress conditions.

The protection of NR was reported in diabetes-induced bone loss in mice through OXPHOS, according to the data of Gao et al. [46]. NR treatment can induce anti-inflammatory signatures for skeletal muscle NAD^+^ metabolism [47]. Sun et al. suggested that NR activates SIRT3 expression for the prevention of peripheral neuropathy [48]. NR can protect mitochondria of the dorsal root ganglion against oxidative stress via the SIRT3/NRF2 pathway. Taken together, this can be used to boost metabolic protection under systemic diabetic conditions. Although NAD^+^ boosting seems beneficial for DR management, more studies are needed to link NAD^+^ metabolism and DR development and progression.

## 4. Clinical Data and Translational Challenges

NR and NMN supplementations are generally safe and well tolerated in humans, although the therapeutic dosage needs more investigation. Clinical studies have shown that its supplementation can substantially elevate NAD^+^ metabolites in blood and peripheral tissues, reflecting improved NAD^+^ bioavailability [49,50,51]. However, the optimal dosage and duration of NMN or NR supplementation for each metabolic disease remain unclear.

As one of the general supplements, NMN is easily purchased without strict restrictions. However, NMN’s potent effects as a drug have been gradually unraveled in many tissues and diseases, including diabetes, aging, obesity, eye diseases, cardiac function, and various neurologic diseases and disorders.

Recently, long-lasting NMN supplements have also been on the market. NMN dose and duration of the administration may need further investigation for the proper efficacy and potential side effects of an overdose.

As SARM1 is a metabolic sensor that can be activated by an increased NMN/NAD^+^ ratio to induce axon degeneration under certain circumstances [52,53], safety issues should be well examined. Therefore, balancing NMN and NAD^+^ levels or indirectly boosting NAD^+^ metabolism is critical to prevent pathological SARM1 activation. Many researchers have aimed to develop promising novel NAD^+^ boosters that remove this side effect. However, those novel drug targets should first be well tested at the preclinical study stage, with efficacy and safety.

## 5. SIRT1, SIRT3, and SIRT6 with Neuronal Protection

SIRTs constitute a group of NAD^+^-dependent deacetylases essential for controlling cellular metabolism, stress reactions, and aging processes. SIRT1, SIRT3, and SIRT6 have become crucial regulators that possess unique yet occasionally overlapping roles [54,55,56]. The NAD^+^-dependent deacetylases, SIRT1, SIRT3, and SIRT6, work synergistically to reduce inflammation, improve metabolic processes, and reduce oxidative stress by reacting to NAD^+^ availability. Their function and coordination across the cells are directly controlled by NAD^+^ metabolism.

SIRT1 has been the best studied among all SIRTs, and it is important for cellular protection. SIRT3 is one of the most prominent deacetylases regulating acetylation levels in mitochondria. SIRT6 improves telomerase activity to protect against DNA damage.

Chaqour et al. recently focused on a gene program related to mitophagy and mitochondrial biogenesis, improving the bioenergetic function of RGCs in a mouse model of optic neuritis resulting from experimental autoimmune encephalomyelitis (EAE). This study emphasizes the importance of RGC degeneration and neuroinflammation for neuroprotection in glaucoma [57].

Mishra et al. summarized the role of SIRT1 in DR progression, showing the therapeutic effects of strategies targeting SIRT1 activation to preserve retinal neurovascular function and to prevent the continuation of the vicious cycle of mitochondrial injury in DR [58]. Adu-Agyeiwaah et al. used gene therapy with AAV2-SIRT1 to increase retinal SIRT1 expression, and its change reverses functional damage in the retina in *db/db* mice [59].

Ban et al. found neuroprotective effects of retinal SIRT3 against acute light-induced oxidative stress in SIRT3 knock-out mice [60]. The data from this group unraveled the critical roles of SIRT3 in photoreceptor neuronal survival and suggested that SIRT3 might be a therapeutic target for oxidative stress-induced outer retinal damage. Shi et al. demonstrated that SIRT3 mitigates high-glucose-induced retinal microvascular endothelial cell damage [61]. In their study, targeting SIRT3 can modulate mitochondrial dynamics through the optic atrophy 1 (OPA1) pathway. This therapeutic concept can be applied to DR management.

Huang at el. focused on SIRT3’s roles in RPE cells and found that SIRT3 expression decreased under high-glucose conditions, leading to ROS production [62]. Under this condition, SIRT3 can activate mitophagy via the FOXO3 pathway and decrease RPE cell death.

SIRT6 has been found to protect RGCs and the optic nerve from glaucoma and aging, as studied by Xia et al. [63]. Taken together, SIRT activation is promising to manage various metabolic diseases and eye disorders. Therefore, NAD^+^ metabolism and SIRT modulation should be well understood to manage DR and/or AMD.

## 6. Natural Products as NAD^+^ Boosters

Natural products are naturally embedded with bioactive and pharmacological activities for the prevention and protection of metabolic diseases. They include phytochemicals, functional foods, and nutrients, exerting anti-inflammatory and antioxidant effects. Therefore, it is important to facilitate their potential as drugs or supplements.

Many studies showed that lutein and/or zeaxanthin intake is beneficial for managing AMD [64]. Wu et al. suggested that higher intake of lutein/zeaxanthin is associated with a long-term reduced risk of advanced AMD, based on the prospective cohort study [65]. Therefore, those two compounds/nutrients might be helpful in slowing the AMD progression.

Angelia et al. suggested the Mediterranean diet as a modifiable risk factor for AMD, using a systematic review and meta-analysis [66]. Furthermore, omega-3 fatty acids can be a protective factor for AMD, based on the prospective cohort and Mendelian randomization analyses by Xue et al. [67]. Dietary omega-3 fatty acids are well known for lessening inflammation in various tissues under various types of stress conditions [68,69,70,71]. Therefore, a balanced diet and supplementation with omega-3 fatty acids could be a good strategy for managing DR and AMD.

Wan et al. found that grape seed proanthocyanidin extract (GSPE) enhances NAMPT expression and NAD^+^ levels in aging mice, and reduces the RPE cellular senescence [72].

Natural products have been investigated for their potential for human health care. However, their NAD^+^ regulatory effects have not been extensively examined in vitro and in vivo. Therefore, screening of various compounds for NAD^+^ boosters might be an intriguing future topic. Curcumin, resveratrol, salvianolic acid B, pterostilbene, EGCG, apigenin, kaempferol, quercetin, isoliquiritigenin, luteolin, ginsenosides, or berberine can be the therapeutic candidate for improving retinal health with NAD^+^ boosting [73].

Curcumin is a polyphenol and has been shown to target various signaling molecules, supporting multiple health benefits [74]. Corneal neovascularization, corneal wound healing, conjunctivitis, glaucoma, cataract, AMD, and DR can be affected by curcumin treatment, based on various research articles [75].

Resveratrol (3,5,4′-trans-trihydroxystilbene) is also a well-known polyphenolic compound in grape and berry fruits [76]. Yuan et al. [77] showed that resveratrol can protect against RGC loss under STZ-induced diabetic conditions in mice through the NRF2-signaling pathway. Peng et al. [78] showed that resveratrol can reduce ROS production and inflammation in STZ-induced DR mice through the SIRT1 pathway. Soufi et al. [79] also found the protective role of resveratrol in DR through the anti-oxidative stress pathways, using male Wistar rats with STZ induction. Therefore, resveratrol can be used for DR treatment.

Berberine is a yellow, solid, naturally occurring benzylisoquinoline alkaloid. Li et al. suggested the protective effects of berberine for human RPE cells against oxidative stress through AMPK activation [80]. The mitochondrial membrane potential was damaged by oxidative stress. However, berberine attenuated this pathology.

Ginseng contains ginsenosides, and various types of ginsenosides have been identified to date. Hu et al. [81] aimed to investigate the effects of ginsenoside Rg3 on AMD and its therapeutic molecular mechanism. They suggested that ginsenoside Rg3 may protect retinal cells against NaIO3-induced damage by reducing mitochondrial dysfunction caused by ROS production. Bian et al. used the combination of ginsenoside Rb1 and Rd to protect the retina against extensive light exposure in Balb/C mice [82]. Chang et al. [83] found that ginsenoside Re protects the retina against photo-mediated oxidative stress. Ginsenoside Re partially antagonizes the pathologic effects of photo-mediated oxidative stress on Müller cells, which is important for retina homeostasis. Lee et al. [84] found that ginsenoside protopanaxadiol protects ARPE-19 cells from chloroquine through the modulation of autophagy and apoptosis. Yang et al. [85] found that *Panax ginseng* Fraction F3 extracted by supercritical carbon dioxide reduces hydrogen peroxide-induced oxidative stress damage in ARPE-19 cells.

Zhang et al. [86] found that apigenin protects murine retinal cells against oxidative stress by regulating the NRF2 pathway and autophagy. They prepared the solid dispersion of apigenin, as apigenin has poor water and fat solubility. The solid dispersion of apigenin upregulates the antioxidant gene expression through the NRF2 pathway and inhibits oxidative-stress-induced retinal damage. Jiang et al. [87] used apigenin and ethaverine hydrochloride for improving the retinal vascular barrier in human retinal microvascular endothelial cells. Furthermore, they used mice to confirm this enhancing effect. Li et al. [88] found that apigenin can reduce epithelial–mesenchymal transition (EMT) in high-glucose-induced RPE cells through the inhibition of CBP/p300-mediated histone acetylation. This potential therapeutic can be applied to suppress retinal fibrosis under diabetic conditions, as RPE’s EMT process is highly involved in the formation of fibrosis [89,90]. Miao et al. found that apigenin reduces diabetic endothelial dysfunction via the NRF2/HO-1 signaling pathway [91].

Quercetin is a member of the flavonoid family, and it is also known as the most prominent antioxidant. Cao et al. [92] found that quercetin can protect ARPE-19 cells from oxidative stress by inhibiting pro-inflammatory molecules and several apoptosis markers. Chai et al. [93] suggested the protective roles of quercetin against diabetes-induced retinopathy in rats through the induction of antioxidant HO-1 expression. Liu et al. [94] found that quercetin protects against retinopathy by regulating the gut–retina axis, enhancing the antioxidant capacity in the retina.

Emerging evidence demonstrated that kaempferol is a strong polyphenol antioxidant that can be found in fruits and vegetables [95]. The beneficial effects of dietary kaempferol are promising in anti-cancer therapy with its strong antioxidant properties. From an ophthalmological point of view, kaempferol treatment can protect the retinal photoreceptor cells from degeneration caused by extensive light exposure in Balb/C mice, studied by Noguchi et al. [96]. This conclusion was supported by histologic and functional evaluations.

Amino acids (including taurine) and vitamins can also be used to boost NAD^+^ metabolism in the retina. Depending on their effects and dosage, they can become a good cofactor involved in NAD^+^ boosting. This aspect is interesting and should be further examined with NMN or NR treatment, depending on the dose and duration of the administration. Vitamin B6 has been known to prevent choroidal neovascularization formation in mice [97], and it also has neuroprotective effects against ischemic retinopathy in experimental models [98]. Therefore, it might be good to try the NMN and vitamin B6 combination’s effects against DR or AMD progression.

Although many natural products and their compounds show promising effects against various diseases, more studies are highly needed, as aspects of the side effects systemically or locally have not been clearly discussed. Taken together, various combinations of natural products and NAD^+^ boosters may have promising therapeutic effects.

## 7. Future Directions

Research should focus on conducting well-structured, large-scale clinical and preclinical studies to confirm the advantages of NAD^+^ supplementation and enzyme inhibition in various experimental groups suffering from retinal conditions. In experimental ophthalmology, there are suitable experimental rodent models for each disease. They can be well-used for further study. Examining dosing schedules, treatment length, and inclusion/exclusion criteria will be essential, depending on the disease model. Integrating NAD^+^ boosters with lifestyle changes, dietary antioxidants, and current standard treatments may produce synergistic effects. For instance, vitamins or amino acids and NMN or NR can be mixed together to have some great potential to cure certain metabolic diseases. Moreover, studies on the synergy between natural bioactive compounds from natural products and NAD^+^ boosters may provide improved defenses against inflammation, oxidative stress, and mitochondrial dysfunction. Creating verified biomarkers for SIRT activity and NAD^+^ metabolism in retinal tissues and systemic biofluids will aid in patient stratification and therapeutic efficacy. Furthermore, for understanding the isoform-specific roles of each compound on retinal neuroprotection, targeted mechanistic studies employing conditional SIRT knockout and overexpression rodent models are essential. Creating novel delivery systems (e.g., nanoparticle encapsulation) designed for precise retinal or subretinal targeting will improve treatment effectiveness and reduce systemic exposure. However, systemic NAD^+^ boosting might be metabolically important at certain points. This should be well-considered. Understanding SIRT interactions (SIRT1-SIRT3, SIRT1-SIRT6, and SIRT3-SIRT6) and metabolic control in retinal health may open promising pathways for disease intervention.

## 8. Conclusions

**Figure 1:**
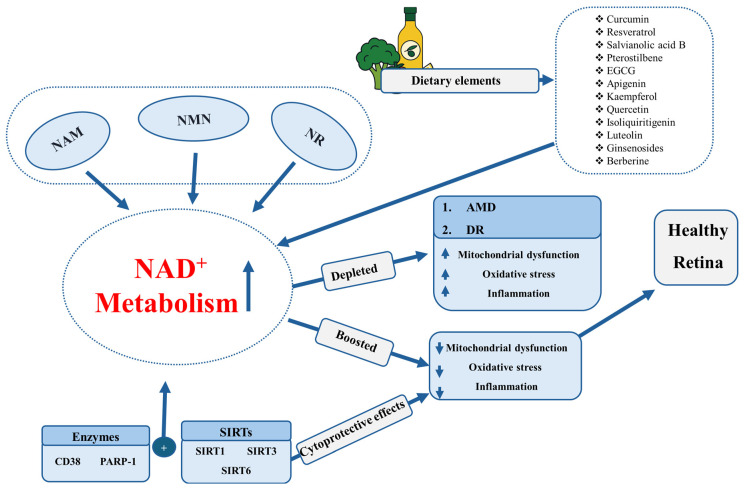
Schematic illustration outlining the role of NAD^+^ metabolism in retinal health and deterioration. NAD^+^ precursors such as NAM, NMN, and NR, along with dietary elements, enhance the production of NAD^+^. Enzymes (CD38, PARP-1) and NAD^+^-dependent SIRTs (SIRT1, SIRT3, SIRT6) control redox balance in cells, maintain mitochondrial stability, and additionally regulate NAD^+^ metabolism. Reduction of NAD^+^ is associated with AMD and DR, characterized by mitochondrial impairment, oxidative stress, and inflammation. These mechanisms can induce choroidal neovascularization/macular degeneration in AMD and retinal neovascularization in DR. More than neovascularization, retinal neuronal cells can also be affected. AMD cases can affect outer retinal neuronal cell death, although there are variations in affected areas (outer, inner, or both). DR cases can affect the inner and outer retinal neuronal and non-neuronal cells. Conversely, enzyme and SIRT-driven cytoprotective mechanisms improve mitochondrial function and decrease oxidative stress and inflammation, thereby preserving retinal health and preventing neurovascular damage. Although combinations of NAD^+^ boosters and other natural product-derived compounds have not been examined for retinal protection, this further direction is one of the promising therapeutic strategies to cure ocular diseases, including DR and AMD. This will be further studied with great interest.

**Figure 2:**
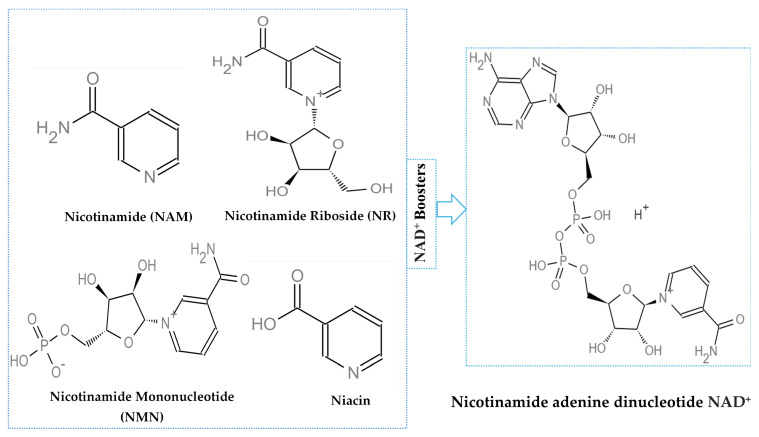
Chemical structures of representative NAD^+^ boosters. Nicotinamide adenine dinucleotide (NAD^+^)-CID 925, Nicotinamide (NAM)-CID 936, Nicotinamide riboside (NR)-CID 439924, Niacin-CID 938, Nicotinamide mononucleotide (NMN)-CID 16219737. Chemical structures are annotated with their common names and PubChem Compound Identifiers (CIDs) for reference (https://pubchem.ncbi.nlm.nih.gov/, accessed on 18 September 2025) [99]. NMN and NR enhance NAD^+^ biosynthesis, having health benefits. NMN is synthesized from NAM by the rate-limiting enzyme, nicotinamide phosphoribosyltransferase (NAMPT). NMN is synthesized from NR through an NR kinase-mediated phosphorylation reaction. NMN is converted into NAD^+^ by NMN adenylyltransferases (NMNATs).

The gathered evidence highlights the critical importance of NAD^+^ metabolism and its regulation in preserving retinal integrity in AMD and DR. Preclinical research consistently shows that providing NAD^+^ precursors or blocking NAD^+^-depleting enzymes can alleviate mitochondrial impairment, diminish oxidative stress, and inhibit inflammatory responses in retinal tissues (Figure 1 and Figure 2). Activation of SIRTs enhances neuroprotection by modulating metabolic and inflammatory pathways. Although promising, NAD^+^ boosters remain largely in the preclinical stages for AMD and DR, with clinical application still in its infancy. A limited number of human trials suggest safety and some functional benefits; however, conclusive efficacy data are lacking. Therefore, rigorous safety and efficacy studies are urgently needed in clinical settings to translate these findings effectively. Nutritional strategies enhance drug therapies, highlighting a comprehensive approach in retinal treatment. In general, NAD^+^-enhancing approaches offer significant potential for maintaining retinal health and delaying degeneration but need thorough clinical assessment to determine effective treatments and long-term safety.

## Data Availability

No new data were created or analyzed in this study. Data sharing is not applicable to this article.

## Abbreviations

The following abbreviations are used in this manuscript:

AMD	Age-related macular degeneration
DR	Diabetic retinopathy
NAD^+^	Nicotinamide adenine dinucleotide
NAMPT	Nicotinamide phosphoribosyl transferase
NMNAT	Nicotinamide mononucleotide adenylyl transferase
NAM	Nicotinamide
NMN	Nicotinamide mononucleotide
NR	Nicotinamide riboside
RPE	Retinal pigment epithelium
CNV	Choroidal neovascularization
anti-VEGF	Anti-vascular endothelial growth factor
RGC	Retinal ganglion cell
BRB	Blood-retinal barrier
HO-1	Heme oxygenase-1
AGEs	Advanced glycation end-products
PKC	Protein kinase C
SIRT	Sirtuin
STZ	Streptozotocin
GFAP	Glial fibrillary acidic protein
ROS	Reactive oxygen species
IP	Intraperitoneal
GSPE	Grape seed proanthocyanidin extract
PDR	Proliferative DR
HIF	Hypoxia inducible factor
